# Effects of white-tailed deer and invasive plants on the herb layer of suburban forests

**DOI:** 10.1093/aobpla/plx058

**Published:** 2017-10-24

**Authors:** Janet A Morrison

**Affiliations:** Department of Biology, The College of New Jersey, Ewing, NJ 08628, USA

**Keywords:** Deer exclosure, invasive addition, *Microstegium vimineum*, multi-group structural equation model, suburban forests

## Abstract

Lack of hunting and predators and proximity to human communities make suburban forests prone to high deer abundance and non-native plant invasions. I investigated these likely drivers of community structure in the herb layers of six suburban forests in one region of New Jersey, USA. In 223 plots I assessed the herb layer response to 2.5 years with or without deer fencing and the early stage of invasion from seed additions of *Microstegium vimineum*, an invasive, annual grass. Non-native plants and herbaceous native plants were affected very little by fencing or *M. vimineum* invasion. In contrast, across all forests the combination of deer access and *M. vimineum* addition had a strongly negative effect on woody native percent cover. Forests differed in overall fencing effects on woody natives; their cover was greater in fenced plots in just three forests, suggesting greater deer pressure in those forests during the experiment. The early invasion by *M. vimineum* was greatest in two of these same forests, but was not influenced by fencing. Multi-group structural equation modelling compared two groups of forests that differed in vegetation abundance and other characteristics. It paralleled the results above and also showed no negative influence of non-native cover on native cover, even in the forests where non-native cover was greater. It identified a positive effect of light level on herb layer plants in the forests with less vegetation, and also revealed a positive effect of soil water potential (SWP) on non-native plants in the forests with more vegetation, which had higher SWP. These suburban forests within a common region varied widely in native and non-native herb layer abundance, the early success of *M. vimineum* invasion and the herb layer’s response to early invasion and protection from deer.

## Teaser

Suburban forests face the dual challenge of abundant white-tailed deer and multiple non-native plant invasions. This paper presents early findings from a field experiment on herb layer responses to the combination of deer exclosure and addition of the invasive grass Microstegium vimineum. Deer and invasive plants may indeed interact as multiple stressors on the native community; woody native plants were vulnerable only to the combination of deer access and invasion, and structural equation modeling indicated a negative effect of M. vimineum on native plants (woody + herbaceous) only in the forests where deer also had a strong negative effect.

## Introduction

The landscape across much of the modern world is rapidly urbanizing, resulting in a vast patchwork of natural areas embedded within the built infrastructure (Brown *et al.* 2005; Theobald 2014; Homer *et al.* 2015). In regions where the natural biome is forest, as in eastern North America, these areas may be termed ‘suburban forests’. They have become important ecosystems in urbanizing areas, containing a large share of many regions’ biodiversity, providing key ecosystem services and offering a vital connection to nature for many people (Hansen *et al.* 2005; Radeloff *et al.* 2005; Aronson *et al.* 2015). However, their ecology remains poorly understood; they are a perfect example of the so-called ‘semi-natural matrix’ that is an important new frontier for ecology (Hansen *et al.* 2005; Agrawal *et al.* 2007).

Suburban forests face multiple stressors that may act together to create a particularly difficult challenge for the native plant community. In this study, I aimed to investigate the joint roles of white-tailed deer (*Odocoileus virginianus*) and non-native, invasive plant species. Suburban forests’ close proximity to human habitation results in a lack of natural predators and little hunting, and their small size and fragmented nature creates ideal habitat for white-tailed deer (Alverson *et al.* 1988; Masse and Cote 2012; Quinn *et al.* 2013). This combination has led to extraordinarily high deer densities in suburban regions (Urbanek and Nielsen 2013). Suburban forests also typically harbour an abundance of non-native plants, many of which are considered invasive (Robertson *et al.* 1994; Ehrenfeld *et al.* 2001; Hansen *et al.* 2005; Morrison *et al.* 2007; Wang *et al.* 2007; Dolan *et al.* 2011; Aronson *et al.* 2015).

Many forest ecology studies have investigated deer or invasive plants alone (Vilà *et al.* 2011; Habeck and Schultz 2015), but relatively few have experimentally tested both together (Waller and Maas 2013; Christopher *et al.* 2014; Dávalos *et al.* 2015a; Johnson *et al.* 2015), and studies in suburban forests are particularly lacking (but see Baiser *et al.* 2008; Aronson and Handel 2011). There are good reasons to think that deer and invasive plants interact as multiple stressors in suburban forests. Deer at very high densities can create unrelenting, chronic stress on many species, especially those they prefer (deCalesta 1994; Weigmann and Waller 2006; Bressette *et al.* 2012). In fact, deer are recognized as a keystone species (Waller and Alverson 1997) with profound influence on forest communities (Russell *et al.* 2001; Rooney and Waller 2003; Cote *et al.* 2004; Abrams and Johnson 2012; Shelton *et al.* 2014). If multiple invasive species are also a major presence, they can create a second stress through the strong competitive effects they may have, for a variety of hypothesized reasons (e.g. Blossey and Notzold 1995; Keane and Crawley 2002; Richards *et al.* 2006; Callaway *et al.* 2008). Additionally, deer may play a key role in plant invasions by depleting the native community and opening niches for unpalatable new arrivals (Relva *et al.* 2010) and by disturbing the forest floor, which can promote establishment of weedy species (Knight *et al.* 2009). In suburban forests, where deer pressure can be exceedingly high, we may expect a particularly important role for deer during plant invasion.

I investigated the combined effects of deer and invasive plants on the herb layer community in a fencing × invasive addition experiment in suburban New Jersey, USA. The herb layer is an important target for study (Gilliam 2007). It contains much of a forest’s plant diversity, including tree seedlings, which show an alarming lack of recruitment in many eastern North American forests (Abrams 2003), often attributed to juvenile displacement by invasive plants or to deer herbivory (Rooney and Dress 1997; De La Cretaz and Kelty 2002). A common herb layer invader of many suburban forests in the mid-Atlantic region of North America is *Microstegium vimineum* (Japanese stilt-grass) (Hunt and Zaremba 1992), which I used in this experiment.

*Microstegium vimineum* is an annual, warm-season grass from eastern Asia that is of serious concern for the conservation of native forest biodiversity in North America (Adams and Engelhardt 2009; Flory and Clay 2010; Simao *et al.* 2010), including in urbanizing landscapes (Vidra *et al.* 2006; Aronson and Handel 2011). Many studies have been conducted on its history and ecology (e.g. Leicht *et al.* 2005; Morrison *et al.* 2007; Eschtruth and Battles 2009a; Marshall *et al.* 2009; Eschtruth and Battles 2014; Flory and Bauer 2014). A handful have considered both white-tailed deer and *M. vimineum* in some manner. They suggest that deer and *M. vimineum* have interactive effects on native plants (Johnson *et al.* 2015), and that deer may facilitate *M. vimineum* invasion (Baiser *et al.* 2008; Eschtruth and Battles 2009b; Knight *et al.* 2009; Johnson *et al.* 2015) by creating disturbed microsites for its establishment (Oswalt and Oswalt 2007) and by reducing competitors via herbivory, while rarely including it in their diet (Averill *et al.* 2016; but see Williams and Ward 2006). These studies, however, were not conducted in suburban forests, employed a small number of deer exclosures and/or relied only on the natural colonization or removal of *M. vimineum*, both of which pose methodological problems (Kumschick *et al.* 2015).

**Figure 1.**
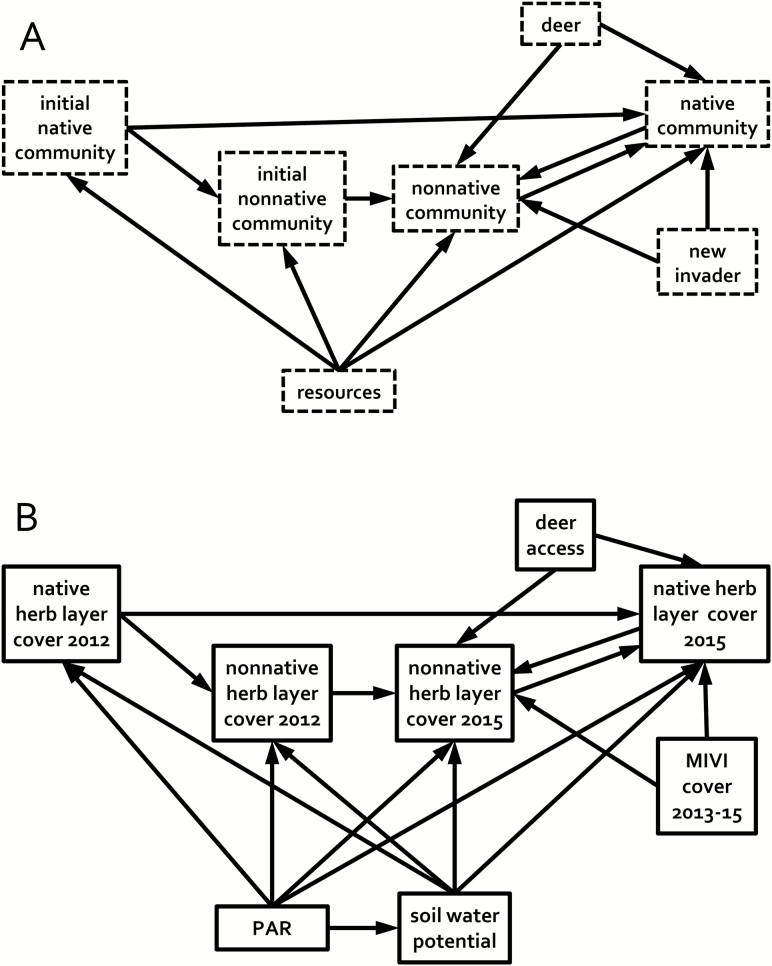
(A) Structural equation meta-model (SEMM), depicting a conceptual framework for how the native and non-native herb layer plant community and resources interact within the forest as a system. It hypothesizes that the native community at the time of study should be affected by its initial state before the experiment began, the existing non-native component of the community, the arrival of new invasive plant species, deer and resources. The non-native community should have similar influences. (B) Initial structural equation measurement model that reflects the SEMM.

The factorial experiment was combined with structural equation modelling (SEM; Grace 2006), which allows for investigation of system-wide responses in an experiment, including direct and indirect effects (Gough and Grace 1999; Tonsor and Scheiner 2007; Lamb and Cahill 2008; Grace *et al.* 2009; Youngblood *et al.* 2009). The experiment and SEM provided tests of the following hypotheses about the roles of deer and invasive plants in suburban forests: (i) deer negatively affect native plants; (ii) *M. vimineum* negatively affects native plants; (iii) deer facilitate the initial invasion of *M. vimineum*; (iv) deer positively affect the non-native plant community in general; and (v) the combination of deer and *M. vimineum* has a synergistic negative effect on native plants. In addition to these specific hypotheses, the SEM tested a system-wide hypothesis as proposed in the structural equation meta-model (SEMM) shown in Fig. 1A (Grace *et al.* 2010; Grace *et al.* 2012).

This experiment focused on the early stage of a new invasion and was based on just 2.5 years of deer exclosure; so, its tests of the hypotheses are limited to those circumstances. Still, factors that promote the initial establishment of invasive plants are important elements of invasive success and impact (Theoharides and Dukes 2007), and the responses of vegetation to deer exclosure over a short time scale can provide insight into the comparative strength of deer effects among sites and taxa.

## Methods

### Study sites

I conducted the experiment in six forests located across a suburban region of central New Jersey, USA, in Hopewell and Princeton Townships, Mercer County. A small city, Trenton, is 9–18 km from the forests, which are all within 24 km of each other. They are 0.3–2.5 km from the nearest housing subdivision, and the contiguous forested areas at the sites range from ~0.5 to 17 km^2^. Forests were selected from sites that had closed canopies consisting of mixed deciduous trees, soils classified as silt loam or loam with slopes ranging from 0 to 12 % (NRCS Web Soil Survey 2017), and had *M. vimineum* present but also large enough areas without natural invasion for setting up the experimental plots. The most recent study of deer density in the region estimated 32 deer km^−2^ in early spring (Hopewell Valley Deer Management Task Force 2014). The estimate was made by experienced surveyors using distance sampling; it likely represents the lowest point of the year for the deer population because it followed winter and the hunting season (limited hunting is permitted on selected land parcels). This is far above the estimated pre-European colonization density of 4 km^−2^, above which deer can strongly affect vegetation (deCalesta 1997; McCabe and McCabe 1997).

The six forests represent a range of conditions found in the region’s woodlands. Deer densities for the specific forests in the region were not available, but Table 1 summarizes other characteristics that may be related to deer: (i) native shrub cover and herb layer native species richness, which decrease with deer overabundance (Rawinski 2008); (ii) presence in the herb layer of *Quercus rubra* and/or *Q. velutina* (red and black oak) the only preferred deer food species (Wakeland and Swihart 2009) that also are common seed-source canopy trees in each of this study’s forests; (iii) deer browse signs; and (iv) hunting history provided by Hopewell Valley Friends of Open Space and Mercer County Parks Department, the owners and managers of these natural areas. Native species richness was obtained from a spring herb layer census in order to detect all species, including spring ephemerals (the method was the same as in fall census used in this study; see below). *Quercus* presence was taken from spring and fall censuses. Canopy tree importance values (sum of relative frequency, dominance and density for a species in a site) for *Q. rubra* plus *Q. velutina* were obtained with standard procedures (Brower *et al.* 1990). I used a ‘forest secchi’ method (from Michael Van Clef, Hopewell Valley Friends of Open Space), to quantify the percent vertical foliage cover of native woody plants in the deer browse zone, 0.4–1.4 m from the ground (Pierson and deCalesta 2015), measured by sighting on a 1 m^2^ board from across the plot. I measured deer browse from 28 June to 15 July in 2013 and 24 June to 1 July in 2015 in two 0.5 × 4 m belt transects per plot, by looking for the presence of tell-tale shredded twig tips (Pierson and deCalesta 2015) on *Acer rubrum* (red maple), *Carya* sp. (hickories), *Fagus grandifolia* (American beech), *Fraxinus pennsylvanica* (green ash), *Prunus serotina* (wild black cherry), *Quercus* sp. (*Q. rubra*, red oak, and/or *Q. velutina*, black oak) and *Rubus allegheniensis* (blackberry). The number of sampled individuals totalled 3526 across all forests.

**Table 1.** Forest characteristics related to deer pressure. All variables except hunting were measured in 40 16 m^2^ plots per forest. Values for shrub cover and species richness are the mean and SE. All data were from 2012, except percent browse was for species that were browsed in 2013 and 2015 (with total sampled plants in parentheses). The canopy importance values (IV) for red + black oak are shown in parentheses, followed by the ranking of the IV in that forest.

Forest	Years of hunting	Percent native shrub cover	Herb layer native species richness per 16 m^2^	# Plots with red/black oak juveniles in spring, fall	Percent browse index
Baldpate (BAL)	12	55.5 (4.08)	22.2 (0.88)	18, 17 (IV = 33.1, #3)	0.54 % (of 1238)
Nayfield (NAY)	5	29.7 (3.94)	12.9 (0.48)	18, 22 (IV = 84.7, #2)	3.75 % (of 799)
Herronton (HER)	17	14.6 (3.40)	20.6 (0.75)	9, 11 (IV = 32.4, #5)	2.69 % (of 605)
Eames (EAM)	5	6.2 (2.71)	7.9 (0.33)	6, 0 (IV = 16.0, #5)	8.53 % (of 215)
Curlis (CUR)	0	2.5 (0.85)	6.8 (0.37)	4, 5 (IV = 94.0, #2)	11.06 % (of 327)
Rosedale (ROS)	0	0.5 (0.42)	8.7 (0.40)	2, 1 (IV = 29.8, #4)	5.34 % (of 342)

### Experimental design

Each forest originally had 40 square, 16 m^2^ plots. The plot size was appropriate for the scale of the questions—large enough to capture a representative sample of the herb layer vegetation, but small enough to minimize non-treatment environmental variation within the plot. It is within the range of plot sizes in other exclosure studies that were included in a meta-analysis showing no influence of plot size on the effects of deer exclusion on understory community variables, including plant cover and diversity (Habeck and Schultz 2015). Plots were arranged on a grid overlaid on the entire study site, with 4 m between plots, and each was surrounded by a 0.5 m walkway and had a 0.5 m walkway down the middle. Some plots were lost prior to the application of the experimental treatments due to Hurricane Sandy; so, the number of plots ranged from 32 to 40 per forest. Each plot was randomly assigned a fencing or no-fencing treatment and a *M. vimineum* seed addition or no-addition treatment, with the number of treatments per forest as equal as possible.

### *Microstegium vimineum* addition

I collected mature *M. vimineum* seeds from a stand in each forest and 12 other nearby sites in October 2012. I mixed them into one pool for storage at room temperature until they were added to the assigned plots between 15 November and 5 December 2012. I used 2.95 g from the pooled seeds (~2420 seeds) for each plot, which was similar to the seeding rate used in a previous addition experiment (Flory and Clay 2010). I mixed the seeds with 75 mL sand for easier distribution, sprinkled the mixture evenly across the plot, and disturbed the leaf litter and soil surface to allow the seeds to settle; I also disturbed the no-addition plots. Based on observations of *M. viminuem* densities in new, small stands in the forests, I aimed for initial cohorts of at least 30 *M. vimineum* plants per m^2^ to begin the experimental invasions, which would be yielded by a 5 % success rate. Previous work with seed collected from the area indicated very high *ex situ* germination rates (pers. obs.); so, an assumption of at least 5 % recruitment *in situ* seemed reasonable.

### Fencing

I installed 2.3 m tall deer exclosure fences from 20 March to 28 April 2013. They consisted of strong, flexible plastic material with a 4 × 4.5 cm mesh, made for deer fences (Deerbusters.com). They were staked to the ground but had three ~10 × 30 cm gaps at ground level on each side to allow entry by small animals. Previous work showed that this type of fencing has no effect on light or wind speed (Morrison and Brown 2004). Fencing can alter the movement of leaf litter, which accumulates against the fence (but in the 0.5 m border); so, I removed this excess twice per year, and also clipped vines that began growing up the fences.

### Herb layer census

I censused the herb layer in each plot from 17 September through 13 October 2012, before the treatments were applied, and from 23 September through 16 October 2015. I also measured *M. vimineum* cover in 2014, from 19 September through 25 October. I divided the plot into 16 squares, dropped a 0.25 m^2^ quadrat frame into each square without looking, and visually estimated the percent cover of each species in the frame, on a scale from <1 %, 1–10 %, >10–20 %, >20–30 % etc. Multiple researchers made estimates together at the start of the census period until their estimates converged. Most plants were identified to species and their native status was checked on the United States Department of Agriculture’s PLANTS database (USDA, NRCS 2017). The cover score was converted to the range’s midpoint, and the 16 values were averaged to provide one cover value per 16 m^2^ plot, for each species. Plants with insufficient characteristics for identification were not included in this study. Their presence and cover was minimal, with range 0–2 and mean 0.27 species per plot, and mean cover 0.13 % per plot.

### Abiotic measurements

I used a 1 m long ceptometer (AccuPAR model PAR-80 by Decagon Devices, Pullman, WA, USA) to measure the percentage of full-sun photosynthetically active radiation (PAR) at ground level. I recorded PAR under cloudless conditions between 10 a.m. and 2 p.m., at the four corners and centre of each plot and in a nearby field. I used the percentage of full-sun because it took multiple weeks to obtain the measurements in so many plots under clear sky conditions. Photosynthetically active radiation varies with date and time; so, the percentage allowed a more accurate comparison among plots measured at different times. Measurements were made from 15 July to 4 September 2013, 5 August to 23 September 2014, and 16 July to 20 October 2015 (before leaf drop). The SEM used the average PAR values from all years in order to integrate the light environment over the course of the study. I measured soil water potential (SWP) with a bench-top SWP meter (model WP4 by Decagon Devices, Pullman, WA, USA) on two soil samples taken on 14 September 2014 from the top 3 cm of soil in each plot.

### Statistical analysis

The herb layer percent cover variables (herbaceous natives, woody natives, woody non-natives, *M. vimineum*) were analysed with generalized linear mixed modelling (Bolker *et al.* 2009) to accommodate skewed data measured over time, using PROC GLIMMIX in SAS/STAT 9.4 software (SAS Institute 2017). ‘Year’ was a repeated factor, with ‘plot(forest)’ as the subject of repeated measures. The models included ‘forest’, ‘fencing’, ‘year’ and, except for the *M. vimineum* cover analysis, ‘*M. vimineum* addition’—and all interaction terms. ‘Plot(forest)’ and all terms with ‘forest’ were random effects. The analyses were conducted with the β distribution and a logit link function, which is appropriate for right-skewed proportion data, and which reduced heteroscedasticity and non-normality in the residuals (Stroup 2015). The R-side covariance matrix for the repeated effect of ‘year’ used the first-order autoregressive covariance structure, AR(1), which accounted for greater correlation among measurements taken nearer in time. Kenward–Rogers denominator degrees of freedom (which may provide fractional values) were used as they are appropriate for repeated measures. Model fit was assessed with a generalized χ^2^ statistic. *Post hoc* contrasts of least-squares means utilized the Tukey–Kramer method for multiple comparisons. Herbaceous non-native plants were uncommon, so were not analysed.

I conducted SEM with the sem function in the ‘lavaan’ package (v. 0.5-20) in R (v. 3.3.1) (R Core Team 2016), using maximum likelihood estimation of parameters based on the data’s covariance matrix and the hypothesized model (Rosseel 2012). I first created a measurement model (Fig. 1B) based on the SEMM from Fig. 1A, using variables that in some manner measured the conceptual variables of the SEMM (Grace *et al.* 2012). They included 2012 and 2015 fall herb layer cover of native plants and of non-native plants; *M. vimineum* cover averaged over 2013–2015 to integrate its effect over the entire time period; the resource variables PAR and SWP, which are critical for *M. vimineum* (Webster *et al.* 2008; Warren *et al.* 2011a, b); and fencing or no-fencing, for deer access. All percent cover variables and PAR were converted to proportions and logit-transformed to adjust for right-skew, using the ‘car’ package in R (v. 3.3.1).

I took a multi-group SEM approach (Grace 2006) to compare the SEMs for two groups of forests. This required first developing a reduced hypothesized model that fit each group alone. This was guided by running the full hypothesized SE measurement model with the group’s data, removing paths that were clearly not supported by the data and inspecting lavaan’s modification indices to determine if additional paths not included in the initial model were needed. All retained paths in the reduced model were significant at *P* < 0.05 in one or both of the groups, but also included two paths that were nearly significant at 0.08 and 0.11, indicating that they could be important in the multi-group model. I then fit this reduced model for the entire data set by group, allowing all parameters to vary freely between groups. Next, I constrained all parameters to be the same between groups, and compared the free and constrained models with a χ^2^ difference test, which showed that they were significantly different. To determine which parameters were responsible for the difference, I did single constraint testing, starting with the most similar path coefficients (Grace 2006). I assessed SE model fit with χ^2^ statistics (*P* > 0.05), root mean square error of approximation (lower 95 % confidence limit [CL] < 0.05), the comparative fit index (>0.90) and the standardized root mean square residual (<0.10) (Kline 2015). Model residuals were tested for multivariate normality using the multivariate Shapiro–Wilk normality test. In all cases it was violated, but was improved with the transformations. Because of this, I also calculated a more robust statistic, the Satorra-Bentler χ^2^ (Satorra and Bentler 1994), and ran the Bollen–Stine bootstrap with 1000 bootstrap replicates to provide bootstrapped *P* values (Rosseel 2012). All results reported here are based on the bootstrap.

## Results

### Herb layer cover

**Figure 2.**
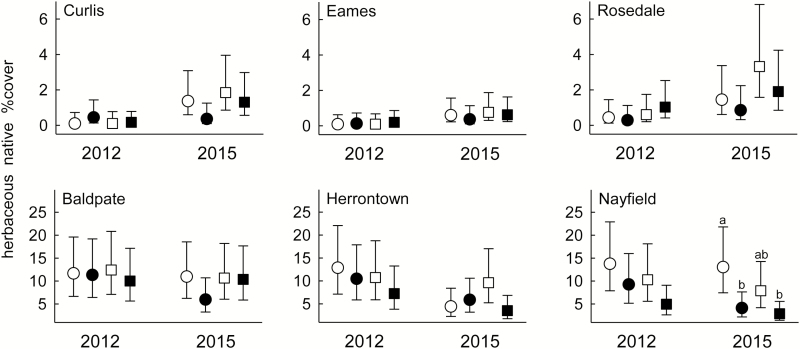
Fall cover (%) of herbaceous native species in 2012 and 2015 within six suburban forests in central New Jersey, in fenced plots (square) or in plots with deer access (circles) with (filled symbols) or without (open symbols) *Microstegium vimineum* seed addition. Data are means ± 95 % CI (back-transformed from logits) of 8–10 plots. Different letters above means indicate significant differences in a forest within a year (Tukey–Kramer adjusted *P* < 0.05).

**Figure 3.**
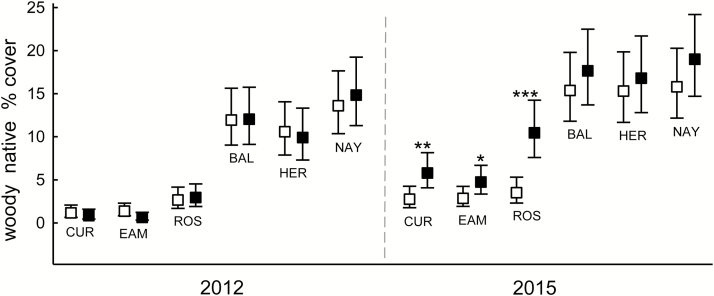
Fall cover (%) of woody native species in the herb layer within six suburban forests in central New Jersey, in fenced plots (filled symbols) or in plots with deer access (open symbols). Data are combined across the *Microstegium vimineum* seed addition and no-addition plots, and are means ± 95 % CI (back-transformed from logits) of 16–20 plots. Asterisks above the mean indicate significant differences in a forest within a year (Tukey–Kramer adjusted: *P < 0.05, **P < 0.01, ***P < 0.001).

Percent cover of native and non-native plants varied among forests, with generally less cover in Curlis, Eames and Rosedale than in Baldpate, Herrontown and Nayfield in both 2012 (pre-treatment) and 2015 (Figs 2, 3 and 5). There were no significant differences within a forest in 2012 between plots that were assigned to different treatments.

**Figure 4.**
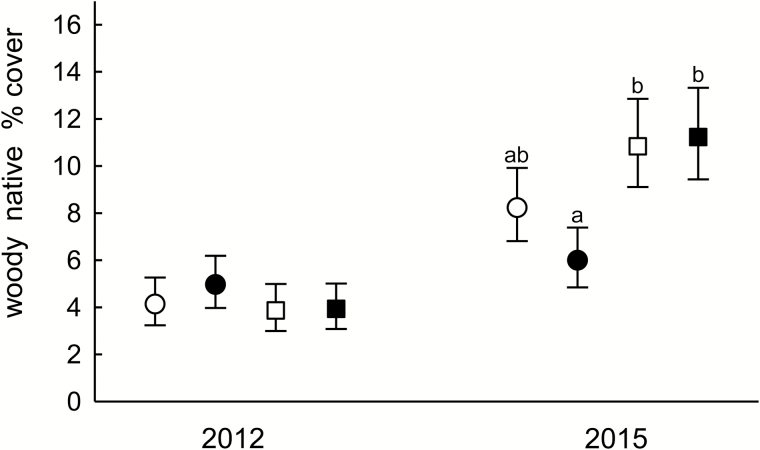
Fall cover (%) of woody native species in the herb layer within six suburban forests in central New Jersey, in fenced plots (square) or in plots with deer access (circles) with (filled symbols) or without (open symbols) *Microstegium vimineum* seed addition. Data are combined across all forests and are means ± 95 % CI (back-transformed from logits) of 55–56 plots. Different letters above the mean indicate significant differences within a year (Tukey–Kramer adjusted *P* < 0.05).

**Figure 5.**
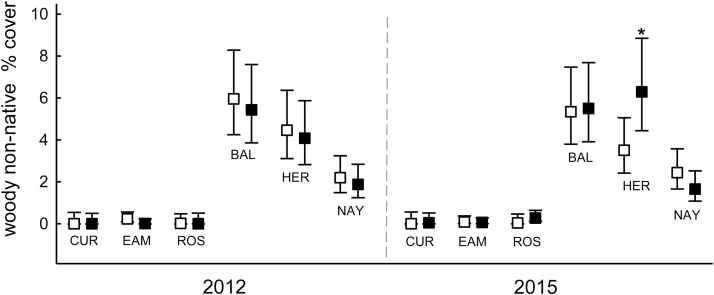
Fall cover (%) of woody non-native species in the herb layer within six suburban forests in central New Jersey, in fenced plots (filled symbols) or in plots with deer access (open symbols). Data are combined across the *Microstegium vimineum* seed addition and no-addition plots, and are means ± 95 % CI (back-transformed from logits) of 16–20 plots. Asterisks above the mean indicate significant differences in a forest within a year (Tukey–Kramer adjusted *P* < 0.05).

Herbaceous native cover did not respond significantly to fencing or *M. vimineum* addition or their interactions in general. However, in Nayfield cover was lower in addition plots (fenced or unfenced) relative to unfenced no-addition plots, leading to a forest × fencing × addition × year interaction (*F*_5, 98.6_ = 5.29, *P* < 0.0001; Fig. 2).

Woody native cover was greater in fenced plots regardless of *M. vimineum* addition, but only in Curlis, Eames and Rosedale, causing a forest × fencing × year interaction (*F*_5, 205_ = 3.63, *P* < 0.01; Fig. 3). Across all forests, woody native cover was lower in unfenced addition plots than in fenced plots with or without addition, creating a fencing × addition × year interaction (*F*_1, 205_ = 11.72, *P* < 0.001; Fig. 4). No other main effects or interactions were significant for woody native cover.

Non-native woody cover was generally not significantly affected by the treatments or their interactions. However, cover increase in fenced plots at Herrontown resulted in a forest × fencing × year interaction (*F*_5, 115.1_ = 2.97, *P* < 0.05; Fig. 5).

### *Microstegium vimineum* percent cover

**Figure 6.**
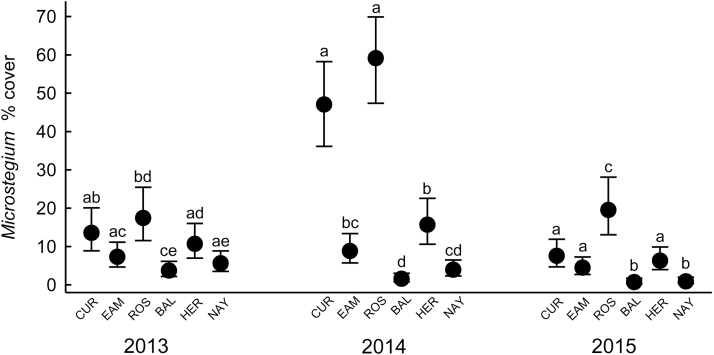
Fall cover (%) of *Microstegium vimineum* in seed addition plots within six suburban forests in central New Jersey. Data are combined across fenced and deer access plots and are means ± 95 % CI (back-transformed from logits) of 16–20 plots. Different letters above the mean indicate significant differences within a year (Tukey–Kramer adjusted *P* < 0.05).

Percent cover of added *M. vimineum* was not affected by fencing but varied greatly among forests and years, causing a significant two-way interaction (forest × year; *F*_10, 244.5_ = 11.62, *P* < 0.0001). Cover in single plots ranged from 0 to 98 %, with the highest mean cover in Curlis and Rosedale, particularly in 2014 (Fig. 6).

### Structural equation model

**Figure 7.**
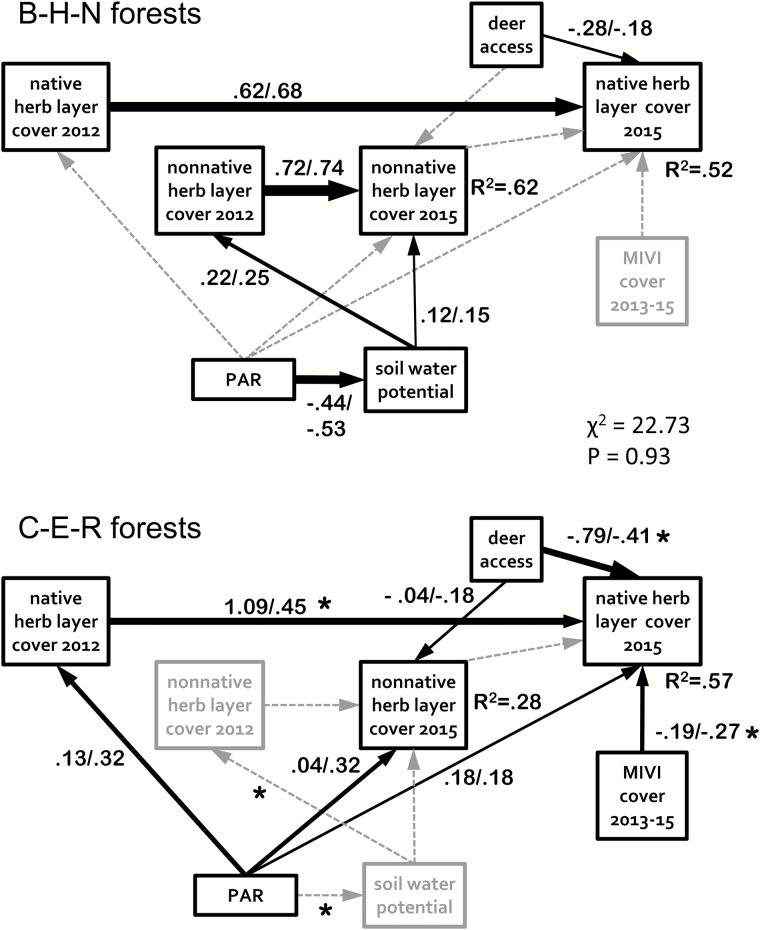
Multi-group SE model, with two groups of forests: Baldpate, Herrontown and Nayfield (B-H-N) and Curlis, Eames and Rosedale (C-E-R). Paths with an asterisk in the C-E-R group contributed significantly to the model-wide difference between the groups. Paths with solid black lines are labelled with unstandardized/standardized path coefficients and are significant in that group at *P* < 0.05. Non-significant paths are shown as dotted grey lines. Widths of the arrows indicate strength of the path based on the standardized path coefficient. *R*^2^ values indicate the proportion of the 2015 cover variables explained by the model. The χ^2^ fit statistic is for the entire multi-group model.

The identification of two groups of forests to compare with multi-group SEM was based on contrasts in their vegetative cover, responses to the experimental treatments by the native woody plants (Figs 3 and 4) and the deer-related characteristics in Table 1. One group consisted of Baldpate, Herrontown and Nayfield (B-H-N) and the other consisted of Curlis, Eames and Rosedale (C-E-R). The modelling revealed both similarities and differences between the groups. The process resulted in dropping six paths that were included in the initial measurement model based on the conceptual model (Fig. 1) but that were not significant for either group, indicating a shared lack of importance for these relationships in both groups of forests. No additional paths were needed to fit the models, and all retained paths were required in order for the model to fit one or both of the groups (see all arrows in Fig. 7).

Three paths that led directly to 2015 native cover contributed significantly to the model-wide differences between the two groups (Fig. 7): (i) deer access negatively influenced native cover in both groups, but more strongly in the C-E-R forests; (ii) *M. vimineum* cover negatively influenced 2015 native cover only in the C-E-R forests; and (iii) 2012 native cover positively influenced 2015 native cover in both groups, but more strongly in the B-H-N forests. One indirect path to 2015 non-native cover also contributed significantly to the group difference. In the B-H-N forests only, PAR had a negative influence on SWP, which had a positive influence on 2012 non-native cover, which had a strong positive effect on 2015 non-native cover. Other paths that were significant in only one group’s model did not contribute significantly to the model-wide difference between the groups.

## Discussion

The results from this study of suburban forests depended on experimental interactions, variation among sites and type of herb layer plant. Deer exclosure and *M. vimineum* addition had no consistent effects as single factors across sites, but in certain combinations they did, even with a limited time frame and generally low *M. vimineum* cover. Understanding the long-term consequences of the experimental treatments will require continuing study, but the results from 2.5 years, along with the SEM, provide some insight into each hypothesis.

The first hypothesis predicted that deer would negatively affect native plants, based on the very high deer density in the region. There was greater native cover in deer exclosures, but only in Curlis, Eames and Rosedale and only in the woody component of the community. The SEM also showed that the negative effect of deer access on native cover was much stronger in the Curlis-Eames-Rosedale (C-E-R) group. The greater effect in these three forests suggests that they experienced greater deer pressure during the experiment, which is consistent with the deer-related forest characteristics in Table 1 and the low native cover in the C-E-R forests shown in Figs 2 and 3. It is possible that their sparse understories are due to factors other than or in addition to deer pressure; so, further efforts to document deer density in the separate forests would be useful.

A recent meta-analysis similarly detected community-level responses to deer exclosures by woody but not herbaceous species (Habeck and Schultz 2015). Given that other, single studies have shown herbaceous responses (e.g. Abrams and Johnson 2012; Begley-Miller *et al.* 2014), the authors of the meta-analysis provided several hypotheses for the difference (from Tanentzap *et al.* 2012), which may apply to this study as well. In particular, herbaceous cover in the C-E-R forests was very low in all plots; so, it may be that a cover response over just 2.5 years of fencing was limited by low energy reserves in perennating organs and/or a lack of nearby seed-producing plants. Indeed, it can take many years for a depauperate herbaceous community to exhibit a response to deer exclosure (McLachlan and Bazely 2001; Royo *et al.* 2010). In contrast, the woody community can respond more rapidly (Aronson and Handel 2011; Shelton *et al.* 2014). The canopy provides a steady supply of tree seeds, and the shrub species in these forests (e.g. *Crateagus* sp., *Lindera benzoin*, *Viburnum* sp.) are bird-dispersed and can be readily introduced from other sites.

Earthworm activity also could have played a role in the lack of an herbaceous cover response to deer exclosure. A pilot study in a subset of 16 plots per forest detected seven earthworm species, including six non-natives. Their abundance varied, with greater totals in Rosedale (=312) and Curlis (=103) compared to the other forests (=3–60) (unpubl. data). A very active earthworm community could be a major force disrupting the herbaceous community (Bohlen *et al.* 2004; Dobson and Blossey 2015), independent of the 16 m^2^ treatment plots and overriding any effect from deer exclosure. However, earthworm abundance in Eames was much lower (=13) than in Rosedale and Curlis, yet it too had low herbaceous cover in both deer access and exclosure plots.

 The second hypothesis predicted that *M. vimineum* addition would negatively affect native plants. This experiment included just the early stage of invasion, and its cover was low in many plots, so a weak effect would be expected. Indeed, herbaceous native cover was significantly lower in addition plots in just one forest, Nayfield. Competition from *M. vimineum* alone also had little to no effect on woody native cover, as shown by the lack of any significant difference between addition and no-addition plots within fencing treatments, across all forests. Other studies have demonstrated competitive effects of dense *M. vimineum* on woody seedlings (Marshall *et al.* 2009; Flory and Clay 2010; Flory *et al.* 2015), with one study showing an effect only when its density was >50 % (Oswalt *et al.* 2007), a level not reached in most plots in this study.

However, woody native cover was significantly lower in plots with both addition and deer access, compared to fenced plots, even at the low *M. vimineum* densities achieved in this experiment. This suggests that woody natives are vulnerable to multiple stresses from *M. vimineum* and deer, even during the early stage of invasion. The SEM suggests further support for their action as joint stressors. It showed a negative path from *M. vimineum* cover to native cover only in the C-E-R forests, the same set of forests in which deer access had the stronger negative path to native cover. These results support the fifth hypothesis, which predicted that the combination of deer and *M. vimineum* has a synergistic negative effect on native plants.

The third hypothesis predicted that deer would facilitate the initial invasion of *M. vimineum* by creating disturbances and depleting potential competitors. The experiment did not offer much support for this hypothesis since *M. vimineum* cover was no greater in deer access plots. Somewhat in support was its greater success in Curlis and Rosedale, two of the forests that perhaps were under greater deer pressure. However, *M. vimineum* cover in Eames, which like Curlis and Rosedale had very low herb layer cover, was generally no greater than in Baldpate, Herrontown and Nayfield. Moreover, the greater earthworm abundance in Curlis and Rosedale may explain, in part, their greater *M. vimineum* cover, given the association between earthworm abundance and *M. vimineum* (Nuzzo *et al.* 2009). These results do not align well with other exclosure studies that have shown increased *M. vimineum* with deer access (Duguay and Farfaras 2011; Abrams and Johnson 2012; Dávalos *et al.* 2015b), but those were done where it was already an established invader and over a longer time period of deer exclosure. Perhaps the early phase of new invasion is less affected by deer.

The fourth hypothesis predicted that deer positively affect the non-native plant community in general. Woody non-native cover, however, was no different in deer access and exclosure plots except in Herrontown, where it was lower with deer access. The general lack of response may be due to the short time of fencing, although 2.5 years was enough for native woody plants to respond in Curlis, Eames and Rosedale. Additionally, the natural non-native cover was much lower in Curlis, Eames and Rosedale, the forests that appeared to have higher deer pressure during the experiment, and the SEM showed a modest negative influence of deer access on non-native cover in the C-E-R group. Most of the non-natives are considered invasive, the most abundant being *Eleagnus umbellata* (autumn olive), *Rosa multiflora* (multiflora rose), *Berberis thunbergii* (Japanese bittersweet) and *Euonymus alatus* (burning bush); so, the results suggest that certain invasive species could be limited by deer in some suburban forests. Other exclosure studies have indicated that non-native plants either did not respond or declined when protected from deer, but there are exceptions (Morrison and Brown 2004; Shelton *et al.* 2014).

The purpose of SEM was to contribute to testing the specific hypotheses above within a system-wide perspective, and also to test the SEMM’s model-wide hypothesis about how suburban forest systems function. Consideration of the proposed paths that turned out to be not significant in the system provides some important insights. First, there was no negative effect of resident non-natives on natives, even in the B-H-N group, where non-native cover reached 10–20 %. I proposed the path in the SEMM on the expectation that invasive non-native plants should negatively affect the native community, but evidence across studies does not consistently show such negative effects (Habeck and Schultz 2015).

Second, natives had no effect on non-natives in the SEM. I proposed this path based on the idea of biotic resistance (Levine *et al.* 2004), whereby non-native plants should do better where there is less competition from natives and more available niche space. This was not the case; so, in these forests other factors likely are more important for non-native success than are their interactions with the native plant community (Gilbert and Lechowicz 2005; Theoharides and Dukes 2007).

Third, other non-natives were unaffected by the amount of cover of *M. vimineum*, the SEMM’s new invader, even in the C-E-R group, where it had higher cover. More time could eventually result in an effect of *M. viminuem* on the non-native community, but in the early invasion of this study there was no effect comparable to that on the C-E-R forests’ native communities. This suggests that non-native plants may be more competitive against a new invader than is the native community.

Fourth, the resources that were included in the SEM—light and water—did not affect all of the vegetation variables as the SEMM proposed. Photosynthetically active radiation had no path to 2012 non-native cover, and SWP had no paths to 2012 or 2015 native cover, suggesting that other factors were more important for these variables.

The paths retained in the SEM but that were different between the two forest groups also revealed important features of these systems. The group differences that are relevant to the specific hypotheses were discussed above, but there were other group differences as well. First, 2015 native cover was more strongly influenced by 2012 native cover in the B-H-N group, reflecting the fact that in the C-E-R group 2015 native cover was also strongly affected by deer access and *M. vimineum* cover.

Second, the roles of resources differed markedly between the groups. Light (PAR) had significant positive paths to vegetation only in the C-E-R group (although none of these paths contributed significantly to the model-wide difference between the groups). The herb layer in those forests received on average 1.6 times the PAR as the other group (4.6 vs. 2.9 %), and it was more variable (coefficient of variation, *CV* 1.15 vs. 0.93). Unlike the C-E-R forests, the B-H-N forests have a developed shrub layer resulting in lower and more uniform herb layer PAR. This difference likely explains why PAR directly influenced the vegetation only in the forests with less shrub cover.

There was a negative path from PAR to SWP in the B-H-N but not the C-E-R group. Greater insolation at the forest floor should result in drier soils; so, it was surprising that the effect was not evident in the C-E-R group, where herb layer PAR was greater. It may be that lower variation of SWP in that group (*CV* 0.55 vs. 0.74) made it more difficult to detect a relationship between variables. Soil water potential had its own direct effects, but only in the B-H-N group. It was higher on average in those forests (−1.11 vs. −1.58 mPa), perhaps due to greater shade from the shrub layer, and so may have greater capacity to influence the vegetation. Only non-native cover was affected, however, with positive paths from SWP to both 2012 and 2015 non-native cover, suggesting that soil moisture is a key resource for plant invasion in these forests.

## Conclusions

This research was aimed at increasing basic ecological understanding of suburban forests, which are faced with the dual challenge of abundant deer and invasions by many non-native plant species. The study offers four main conclusions. (i) Multiple-site studies are needed. The observational and experimental results were quite different among the set of six nearby suburban forests. (ii) Forests with very depauperate understory vegetation, like Curlis, Eames and Rosedale, may particularly benefit from deer management. The future trees and shrubs in the herb layer of these forests responded positively to just 2.5 years of protection from deer. (iii) Deer and *M. vimineum* may interact as multiple stressors on the herb layer community in some suburban forests. Woody native plants were vulnerable to the combination of deer access and *M. vimineum* addition, and the SEM showed a negative effect of *M. vimineum* cover on the native herb layer in the set of three forests where deer access also had a negative effect. (iv) The abundance of non-native, invasive plants among the six forests also was highly variable and followed the pattern of native abundance, being very low in the three depauperate forests. If these forests are indeed under more severe deer pressure, this pattern suggests that deer could be an important driver of both native and non-native plant community structure in suburban forests. To address this hypothesis, additional multisite research is needed in suburban forests with a range of known deer densities, histories and associated deer pressures.

## Sources of Funding

This work was supported by two grants from the National Science Foundation to J.A.M. (DEB 1257833, DEB 0933977); The College of New Jersey’s (TCNJ) Barbara Meyers Pelson ‘59 Chair in Faculty-Student Engagement; TCNJ’s Support for Scholarly Activity Award, Sabbatical Award and the Mentored Undergraduate Summer Experience.

## Conflicts of Interest

None declared.
